# Effects of immediate and distant health consequences: different types of health warning messages on sweets affect the purchase probability

**DOI:** 10.1186/s12889-023-16760-y

**Published:** 2023-09-30

**Authors:** Clara Mehlhose, Antje Risius

**Affiliations:** 1 Marketing of Agricultural and Food Products, Department of Agricultural Economics and Rural Development, University of Göttingen, Platz Der Göttinger Sieben 5, 37073 Göttingen, Germany; 2 University of Education Schwäbisch Gmünd, Institute for Health Sciences, Oberbettringer Str. 200, 73525 Schwäbisch Gmünd, Germany

**Keywords:** Health warning labels, Graphic warnings, Obesity, Overweight, Diabetes, Purchase probability

## Abstract

**Background:**

Several health control policies have been discussed as a regulatory approach to tackle the increasing prevalence of obesity and other health risks related to sugar consumption. Health warnings, like the ones used in tobacco control worldwide, are one of the most promising approaches. However, in the case of health warning messages for food products, it is much more complicated and involves much more consumer involvement than tobacco guidance. Therefore, it is important to better understand the efficacy, evaluation, and reactance of health warning labels in the food sector regarding consumers’ behavior, persuasion, and perceptions. The aim of this study was to examine how different types (design and message) of health warning messages in combination with graphical applications affect consumer behavior.

**Methods:**

In a 3 × 3 × 3 symmetrical design, 1,040 German participants completed an online discrete choice experiment including various text-only and image-and-text health warning labels on sweets. An accompanying questionnaire assessed socio-demographic variables as well as psychometric scales to understand the relationship between fear, control, reactance, and shocking/inhibiting/mediating health-related warnings.

**Results:**

Our results suggest that especially emotional graphical images combined with text health warning labels might be more influential. The health effects of immediate (caries) and more distant health consequences (diabetes/obesity) differ in their impact. Further, results show that especially when consumers engage in a danger control process for overweight, warning messages have a negative impact on their choices.

**Conclusion:**

Hence, warning labels on sweets can potentially be a decisive factor when communicating health threats related to excessive sugar consumption. In the context of a targeted health policy, we see the need for further research, especially concerning the perception and understanding of noncommunicable diseases (NCDs) in the population.

## Background

Despite rising attention, the worldwide prevalence of obesity and overweight and their health effects remain a global epidemic [1]. The consequences of nutrition-related misbehavior are a leading risk factor for noncommunicable diseases (NCDs) such as diabetes, coronary heart diseases, cancers, or chronic respiratory diseases [2, 3]. Altogether, NCDs are responsible for 71% of all deaths globally (natural deaths plus deaths induced by unhealthy lifestyles) [2, 3]. The large number of people with health complications as a direct consequence of overconsumption has developed into a policy problem of economic significance, as it is very cost-intensive to the healthcare systems [4].

Therefore, different Front-of-Packaging (FOP) nutrition labeling schemes are repeatedly being discussed as a regulatory approach to reduce the purchase and/or consumption of unhealthful products [5–7]. Health warning messages (HWM) are one example for this, aiming to highlight the unhealthfulness of products resulting from their high amounts of key nutrients (e.g., sugar, calories).

As such, health warning messages are intended to enhance better-informed dietary choices, e.g., reducing the consumption of nutrient-poor and high-caloric food and beverages by being present at the exact moment when food choice decisions are made [8, 9]. They stimulate consumers’ cognitive understanding by providing information about the health consequences associated with extensive consumption, which can intensify the intention to change this behavior and reduce purchase intentions for unhealthy food products [10–13]. Additionally, HWM disrupt unconscious influences on food decisions, e.g., food cues and other external triggers, which can lead to a healthier food decision-making behavior [8, 9].

Worldwide, there are manifold experiences from various areas regarding the effectiveness of different types of warning label approaches: Best known and for some years now also widely used are warning labels on tobacco products: their graphical additions underline the textual warnings and are known to have a greater influence on consumers’ behavioral change compared to text-based warnings [14, 15]. As such, they represent an impactful and cost-effective alternative for communicating smoking-associated health risks to consumers [16]. However, the food context is more difficult to judge – both content-wise as well as emotionally. The reason for this is mainly that tobacco is one substance to be addressed; for which the negative consequence can clearly be detected and traced back. Nutrition, however, is a multidimensional activity in which several lifestyle factors have an influence on the health impact. It is not only specific foods or nutrients, but also various other life circumstances, e.g., physical activity and dietary patterns overall that influence food behavior. When it comes to warning approaches in the food sector, Chile was the first country worldwide to implement a mandatory nutrition warning system evidencing ‘high’ levels of calories, sugar, saturated fat, and sodium [17]. Until now five countries (Chile, Israel, Mexico, Peru, and Uruguay) introduced or are working on introducing nutrition warnings [18]. The Chilean warning signs are black octagonal text-based warning signs, that are included on food products’ packages for each of the nutrients (calories, sugars, saturated fat and sodium) that exceed the defined limit value established by the Ministry of Health [17]. To a certain degree, these types of warnings seem to be efficient at discouraging the choice of unhealthy foods [19–24].

Nutrition warnings can be seen as antecedents of health warnings in the food domain, but are not the same. While the literature on the topic of health warning labels on tobacco and alcohol products is extensive, there is a lack of empirical research to better understand the efficacy, evaluation, and reactance of various health warning formats in the food sector regarding consumers’ behavior, persuasion, and perceptions; thus, the impact on consumers’ eating and drinking behavior remains largely unexplored so far [25, 26]. To date, there is only a small number of studies that have applied HWM to eating and drinking behavior, with the majority focused on sugar-sweetened beverages (SSB) [7]. A meta-analysis of these studies provides evidence that HWM on SSB are effective in the sense that they reduce both consumption and purchasing behavior; in addition, they cause stronger negative emotional reactions and increase consumers’ thinking about the potential health effects of SSB [18]. The small number of studies using HWM on snack foods found that these labels can reduce purchase and consumption intentions as well [7, 27–29] and that they increase dietary control and motivation to change eating behaviors [8, 9, 21, 25, 30, 31].

Hence, to get a better overview of how health warning labels could interact in the food sector, different health warning messages (expressing immediate and more distant health consequences) and different types and designs of (graphical) HWM need to be understood.

The aim of this study was therefore to examine how different types of health warning messages in combination with graphical applications affect consumer choices of sweets. Specifically, the effects of different health warning message designs (various text-only as well as image-and-text health warning labels) and different types of warning messages (conveying immediate (caries) or more distant health consequences (diabetes, overweight) were evaluated. By applying the Extended Parallel Process Model (EPPM), it was specifically aimed to differentiate emotional responses and assess their effectiveness in order to guide and relate health communication processes. Overweight, diabetes, and caries served as the utilized health consequences on the labels as they are all associated with extensive sugar consumption [32]. Further, a large number of people directly associate these diseases with sugar consumption [33]. For the graphical HWM, we chose two different types: One had the red road traffic stop sign as graphical information. The other one used shocking pictures similar to those displayed on cigarette packages. Both types of graphical messages were supplemented by textual information about the diseases.

### The Extended Parallel Process Model (EPPM)

In this study, the Extended Parallel Process Model (EPPM) was selected as the theoretical framework to evaluate whether consumers experience fear or danger control processes when exposed to the health-related consequences on the health warning messages and whether these processes influence their purchase intentions.

The EPPM is one of the most common theories to explain the effect of fear appeals, which are “persuasive messages designed to scare people by describing the terrible things that will happen to them if they do not do what the message recommends” [34]. The model has already served as a theoretical basis for a variety of studies [35–39] and is particularly suitable in the context of health communication, as it can be used as a theoretical guideline to evaluate message effects before campaigns [40].

Such an appeal initiates several processes: At first, the potential health threat is evaluated with regard to its severity and the person’s own susceptibility to the threat. The aim is to assess the seriousness of the health threat and the likelihood that it will happen to the consumer personally. These two constructs form the perceived threat. If the perceived threat is evaluated as small, no further reaction will be initiated, and the warning will be ignored (no response). The second validation phase begins under the condition that the threat is perceived as serious, which will result in the person feeling fear. During this phase, the consumer evaluates the perceived efficacy. The efficacy is composed of response efficacy and self-efficacy. The response efficacy describes the effectiveness of the recommended response in preventing the health threat, whereas the self-efficacy indicates whether the individual is capable of realizing the response [40].

Subsequently, two responses can be triggered: When the efficacy is perceived as greater than the threat, it causes the initiation of *danger control processes*. During this process, people are motivated to control the potential danger by weighing up strategies to prevent the threat [34]. Conversely, when threat outweighs efficacy, people engage in *fear control processes*. When this occurs, individuals do not believe in their own abilities to avert the threat; they are controlled by fear. Besides ignoring the health threat, people engage in reactance, issue derogation, or perceived manipulation [41].

## Materials and methods

### Experimental design and participants

The design followed a cross-sectional within-subject online survey design procedure. The data collection consisted of two empirical assessments, an experimental part (= choice experiment) and a survey part, which were both part of one online questionnaire. Both parts will be described in detail below.

The participants were approached through a market research agency (Respondi AG, Köln). The questionnaire was answered by 1,105 respondents over 18 years of age. Quotas were set in terms of gender, age, level of education, and net household income to generate a sample approximately representative of the German population in regard to the selected characteristics. The data was collected in October 2019.

### Choice experiment

A symmetrical design with 3 (SYMBOL: Shock, Stop, No symbol) × 3 (WARNING: Diabetes, Overweight, Caries) × 3 (PRICE: High, Medium, Low) conditions was used for this study. As another alternative, a “no-buy” option was integrated in case a person did not want to choose one of the alternatives. The no-buy option is usually integrated as a control option. It statistically serves to capture the disambiguity and non-preferences, which are to be assessed just as well as the preferences for selective quality criteria. The attributes and the associated levels are shown in Table 1.

**Table 1:** Health warning label attributes and the associated levels

Attribute	Attribute level
Warning (= health consequences)	Diabetes, Overweight, Caries
Symbol	Shocking picture, Stop sign, No symbol (= text only)
Price	2 EUR (high), 1.5 EUR (medium), 1 EUR (low)

**Fig. 1:**
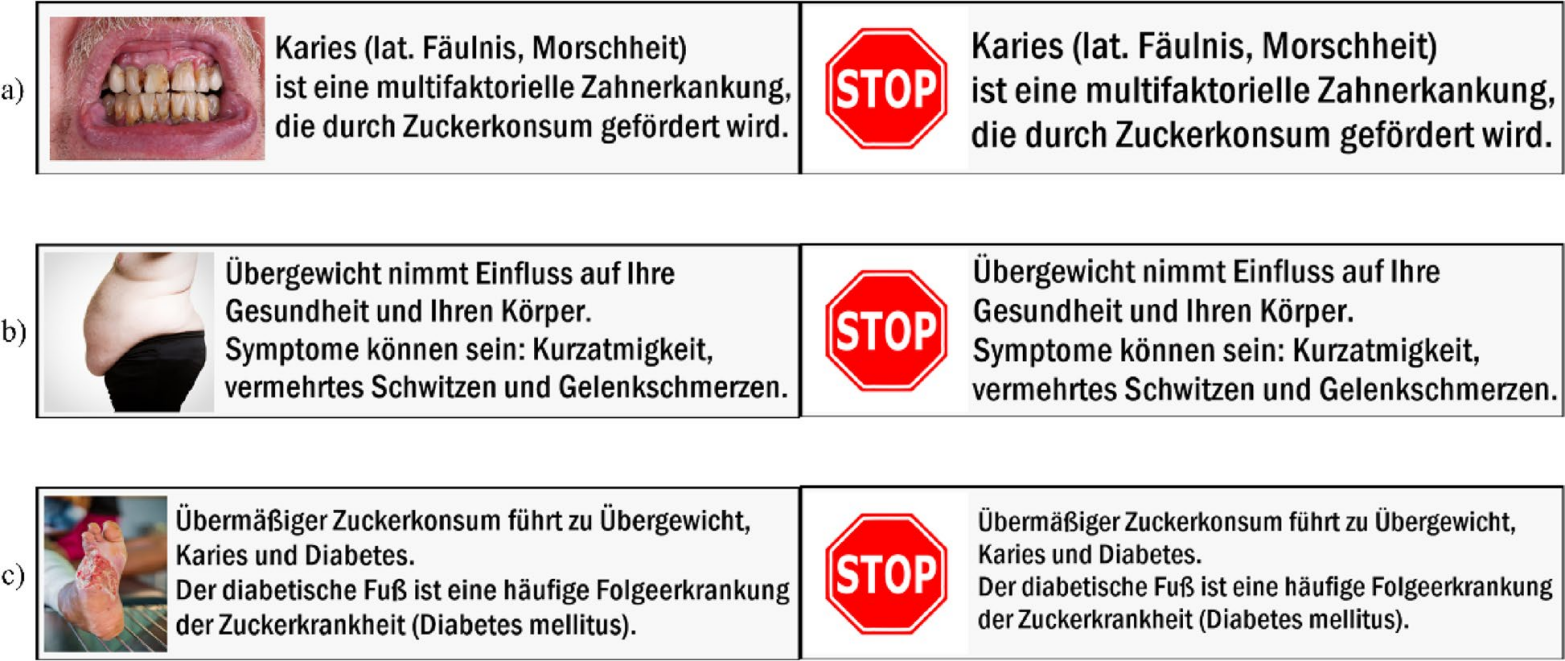
Original health warning labels used in this study, illustrating in each case “shocking picture” and “stop sign” with regard to the textual information about caries (**a**), overweight (**b**) and diabetes (**c**). Translated from the German original the textual information reads: **a** *“Caries (lat. rottenness, morass) is a multifactorial dental disease that is promoted by sugar.”*, **b** *“Excessive sugar consumption leads to tooth decay, obesity, and diabetes. The diabetic foot is a common secondary disease of diabetes mellitus.”*, **c** *“Overweight affects your health and body. Symptoms may include: shortness of breath, increased sweating, and joint pain.”*

Regarding the levels of the associated health consequences, the so-called “WARNINGS” were “caries”, “diabetes”, and “overweight”. They were illustrated as text components next to the graphical symbols (see Fig. 1 for text and graphics). The information about caries read (translated from the German original): *“Caries (lat. rottenness, morass) is a multifactorial dental disease that is promoted by sugar.”* Similarly, the information about the diabetic foot syndrome read: *“Excessive sugar consumption leads to tooth decay, obesity, and diabetes. The diabetic foot is a common secondary disease of diabetes mellitus.”* The information about overweight read: “*Overweight affects your health and body. Symptoms may include: shortness of breath, increased sweating, and joint pain.*”

The levels for the attribute “SYMBOL” were “shocking picture”, “stop sign”, and “no symbol”. Two different types of graphical symbols were used: One used the red traffic stop sign as a graphical symbol (“stop sign”). The other used shocking pictures derived from those used on tobacco products. The “shocking pictures” were considered to show the consequences that were addressed in the textual information about the health consequences (“WARNING” level). Respectively, besides carious teeth, a picture of a diabetic foot or an overweight body was displayed. For the “no symbol” level, only a textual warning without additional graphical symbol was used. Figure 1 shows six exemplary health warning labels with graphical symbols. The levels for the “PRICE” attribute were derived from a customary average price (1.50 EUR), as well as a slightly lower (1 EUR) and a slightly higher price (2 EUR). The price levels were chosen based on a pre-market survey taken prior to the consumer survey.

**Fig. 2:**
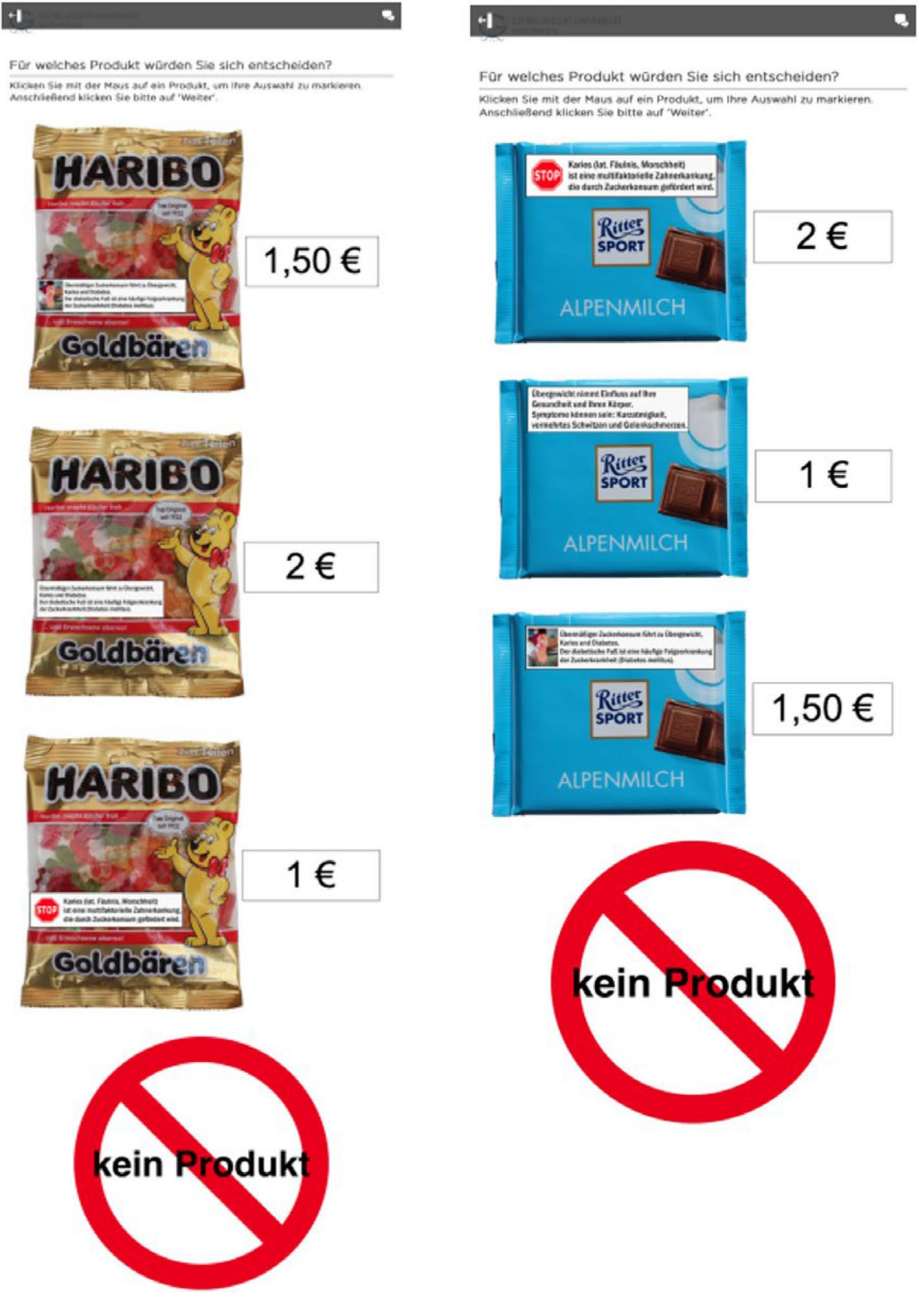
Example of the choice sets: One time the attributes were presented on chocolate, one time on gummy bears, in rotating order. The question was: “Which product would you choose?” The “no‑buy” option was always presented last

A d-efficient discrete choice design was created using the software NGene 1.2.1, accounting for priors by [42]. The discrete choice experiment setting was supposed to remind the respondents of a choice situation available at a vending machine. To make the stimuli situation more realistic, two products (gummy bears and chocolate) served as a medium to display the various HWM (refer to Fig. 2). Both are among the most frequently consumed sweets and snacks in Germany [43] and can be assigned to the category of sweets, are calorie-dense, contain a high amount of sugar and are often found in snack vending machines.

Each respondent was shown a total of 12 choice sets which in each case included 3 product alternatives as well as a no-buy option. Participants received six choice sets with chocolate as the product option and six choice sets with gummy bears. Alternatives in each choice set were presented in a randomized order; however, the no-buy option was always presented at the bottom. Participants were given the following instructions: “Now please imagine the following situation: You have an appetite for something sweet and then decide to buy something from a snack machine. The snack machine contains a variety of different products. On the next page, you are repeatedly presented with 3 products and a ‘no-buy’ option to choose from. Choose between the displayed products and the ‘no-buy’ option if none of the products appeals to you.” Respondents then saw a picture of a vending machine with different snacks, followed by the choice sets as displayed in Fig. 2.

### Questionnaire

The Choice Experiment was integrated into the online questionnaire so that the questionnaire consisted in total of seven main parts: socio-demographic factors, health-related behavior (e.g. consumption of sweets and snacks, physical activity, smoking behavior, etc.), the discrete choice experiment, perception of health warning messages (items in accordance to [44]), theory-related items for EPPM, purchase and consumption behavior and a diet part (e.g. diet style, nutritional knowledge, BMI). Attention check questions were included in the questionnaire to improve data quality.

Following [41], the EPPM dimensions of the perceived threat and the perceived efficacy were measured using respectively six items on a 5-point Likert-scale ranging from “strongly disagree” (1) to “strongly agree” (5). The items were translated into German, and diabetes, caries, and overweight were inserted as health threats. As an example, the items for diabetes are shown in Table 2.

**Table 2:** Exemplary items of the EPPM for diabetes

Construct	Dimension	Item
Perceived threat	Perceived severity	1. “I believe that diabetes is severe.” 2. “I believe that diabetes is serious.” 3. “I believe that diabetes is significant.”
	Perceived susceptibility	1. “I am at risk of getting diabetes.” 2. “It is likely that I will contract diabetes.” 3. “It is possible that I will contract diabetes.”
Perceived efficacy	Self-efficacy	1. “I am able to forgo sweets to prevent getting diabetes.” 2. “Forgoing sweets is easy to do to prevent diabetes.” 3. “Forgoing sweets to prevent diabetes is convenient.”
	Response efficacy	1. “Forgoing sweets works in preventing diabetes.” 2. “Forgoing sweets is effective in preventing diabetes.” 3. “If I forgo sweets, I am less likely to get diabetes.”

### Modelling and data analysis

Descriptive statistics were used for the basic characteristics of the sample and the items of the EPPM theory. The analysis of these results was performed with SPSS (version 27).

A principal component analysis (PCA) was conducted to reduce 16 attitudinal statements concerning participants’ perception of the HWM to three factors using a varimax rotation. The concrete items concerning participants’ perception of the HWM can be seen in Table 5. Eigenvalues and scree plots were used to determine the quality of the factors. Items with low factor loadings (< 0.4) were neglected. The KMO-value (Kaiser–Meyer–Olkin-value) with 0.884 was meritorious [45], the Bartlett test for sphericity was significant with a p-value < 0.001, and the explained total variance was 58.3%. Two items were omitted due to internal inconsistency (the CRA (Cronbach’s Alpha) was poor and < 0.4). The CRAs for all three factors were all > 0.45 (CRA: factor 1 = 0.487; factor 2 = 0.759; factor 3 = 0.617).

To determine if participants engaged in danger control processes or fear control processes, the EPPM items for threat and efficacy were added up and standardized, yielding a threat score and an efficacy score. Subsequently, the threat score was subtracted from the efficacy score, to define whether participants experienced danger control or fear control processes with regard to the presented HWM. A resulting positive score indicates that the person undergoes danger control processes, which means they can control the fear. The most obvious way of preventing the threat would be to not buy the product or, in the case of the choice experiment, to select the no-buy option. A negative score in turn means the person engages in fear control processes, which means they are controlled by fear [41]. One possible reaction would be that respondents reluctantly decide for a choice, despite all the warnings, and ignore all consequences presented on the health messages.

To assess the impact of the HWM on the purchase, the choice experiment was analyzed with a multinomial logit (MNL) model (package mlogit) using the software R (version 4.2.0). The attributes (price, warning, symbol), the compounds from the factor analysis, as well as the EPPM interactions, were included as individual-specific components in the choice model. The no-buy option was modeled as an alternative specific constant (ASC) and was estimated as a fixed alternative.

## Results

The following results section is divided into two parts: 1) the description of the sample, followed by the factor analysis and the results of the EPPM, and 2) the results of the choice model and the measured interactions with respondent characteristics and the EPPM.

### Sample description

The sample consisted of 1,105 datasets, and after data cleaning 1,040 datasets remained for data analysis. Exclusion criteria were response times that were less than half of the median response time (less than 8.2 min) (*n* = 36), participants who did not (correctly) mention their body weight or height (e.g., height over 2.5 m) (*n* = 25), and participants who showed straightlining behavior in their responses (*n* = 4). The average respondent age was 51.7 years. The average BMI of the sample was 27.5 (SD = 6.3).

As quotas were set for gender, age, education, and income, the socio-demographic distribution of the conducted survey is comparable with the general socio-demographic situation in Germany and can therefore be considered approximately representative of the German population in relation to these characteristics. The characteristics of the sample are illustrated in Table 3.

**Table 3:** Socio-demographic characteristics of the sample (*n* = 1,040) compared to the German population (Federal statistics)

Variable	Description	*n*	Sample (%)	German population (%)
Gender	Male	516	49.6	49
	Female	523	50.3	51
	Non-binary	1	0.1	—
Age (years)	(Ø; SD, [min, max])		(51.7; 16.4, [19, 92])	
	18 to 25	81	7.8	9
	26 to 40	215	20.7	22
	41 to 64	455	43.8	44
	> 65	289	27.8	25
Education	No certificate of secondary education	401	38.6	38
	General certificate of secondary education	268	25.8	26
	Higher school certificate or equivalent	371	35.7	36
Net household income	< 1,300 EUR	266	25.6	26
	1,300–2,599 EUR	416	40.0	40
	2,600–5,000 EUR	284	27.3	27
	> 5,000 EUR	74	7.1	7
BMI	Underweight (< 18.5 kg/m^2^)	29	2.8	2^a^
	Normal weight (18.5–24.9 kg/m^2^)	373	36.3	45.3^a^
	Overweight (25–29.9 kg/m^2^)	339	33.0	36.4^a^
	Obese (> 30 kg/m^2^)	287	27.9	16.3^a^

Based on Federal Statistical Office (2018)

^a^no quotas were set for BMI; the data refers to representative data for Germany in 2017 [46]. “n” represents the number of observations. “%” refers to the percentage of the total sample

Some additional questions were asked in the questionnaire to collect basic information about the participants’ consumption and movement behavior. More than 55.8% of the respondents reported to eat sweets at least 2 times a week as a snack (47.8% as reward), while this only applied to 13.61% of the German population with regard to chocolate bars (8.44% for wine/fruit gum) [43]. Also, 58% mentioned that they often or almost always pay attention to the food’s sugar content when eating. The results are summarized in Table 4.

**Table 4:** Participants’ consumption and movement behavior

Characteristic	Description	Percentage
Special type of diet^a^	Vegetarian	4.5
	Vegan	1.3
	Flexitarian	8.4
	Paleo diet	1.7
	Clean food diet	1.8
	Sugar-free diet	5.7
	Weightwatchers	1.2
	Low-carb	5.2
	No special diet	58.4
Eating sweets as a snack	Never	4.2
	On special occasions	5.1
	Once in a month or less often	8.4
	About twice a month	3.8
	About three times a month	4.6
	About once a week	18.2
	About two/three times a week	33.8
	Daily	22.0
Eating sweets as a reward or for pleasure	Never	9.6
	On special occasions	5.8
	Once in a month or less	9.2
	About twice a month	5.4
	About three times a month	4.1
	About once a week	18.1
	About two/three times a week	31.5
	Daily	16.3
Pay attention to sugar content when eating	Never	16.1
	Rarely	25.8
	Often	36.9
	(Almost) always	21.1
Easy physical activity (non-sweating to slightly sweating)	Never	6.0
	< 1 h/week	11.1
	1–2 h/week	21.6
	> 2 h/week	61.3
Medium physical activity (slightly sweating)	Never	15.6
	< 1 h/week	22.3
	1–2 h/week	30.3
	> 2 h/week	31.8
Strong physical activity (heavily sweating)	Never	39.4
	< 1 h/week	25.8
	1–2 h/week	15.4
	> 2 h/week	19.3

^a^multiple answers were possible for the question about the special type of diet. Reported here are only the most relevant answer options

### Principal Component Analysis (PCA)

A PCA was conducted to reduce the attitudinal statements concerning participants’ perceptions of the HWM. The first component that could be extracted was named “ineffectiveness of warning labels”, the second one “helpfulness of warning labels”, and the third one “uselessness of warning labels”. Items and factor loadings of the respective three components are presented in Table 5.

**Table 5:** Items and factor loadings of the factor analysis

Item	F1 = Ineffectiveness of warning labels	F2 = Helpfulness of warning labels	F3 = Uselessness of warning labels
When it comes to products with warning labels, I think twice about whether I really need them		0.849	
The warning labels stir my guilty conscience		0.828	
The warning labels help me because they show the consequences of unhealthy consumption		0.798	
I find the warning labels helpful because they make the dangers of sugar consumption tangible		0.751	
The warning labels would have an impact on my buying behavior		0.749	
I like the warning labels		0.637	
I find the warning labels really disgusting and they put me off		0.623	
I do not pay attention to the warning labels		− 0.606	
The warning labels remind me of cigarette packaging	0.823		
The warning labels will quickly lose their effect as you get used to them	0.566		
The government is trying to exert more and more influence, but not with me!	0.508		
I take care of my health, so the warning labels do not scare me			0.793
I don’t need warning labels because I shop very consciously			0.748
I know the ingredients of the products and make my purchase decision based on them			0.663
**Variance explained**	12.45%	37.21%	8.65%
**Cronbachs Alpha**	0.487	0.759	0.617

### Extended Parallel Process Model (EPPM)

Regarding the danger control process of the three diseases, the highest proportion of participants was engaged in a danger control process for caries (52%), although the difference to overweight (50.2%) and diabetes (48.3%) was very small (Table 6). A total of 259 respondents were experiencing danger control for all three diseases, meaning that they could control their fear of all presented diseases. On the other hand, almost 50% of all respondents were experiencing a fear control process for at least one of the diseases. A total of 269 participants demonstrated fear control for all three diseases, meaning that they were controlled by fear concerning the respective diseases.

**Table 6:** Descriptive results of the danger (DC) and fear control (FC) processes for caries (CA), overweight (OW), and diabetes (DB) in relation to age, gender, and BMI of the participants

Variables	DC CA	FC CA	DC OW	FC OW	DC DB	FC DB	DC all	FC all
n (%)	519 (52%)	479 (48%)	502 (50.2%)	498 (49.8%)	482 (48.3%)	515 (51.6%)	259	269
Mean/SD	0.87 ± 0.73	− 0.94 ± 0.75	0.97 ± 0.76	− 0.45 ± 1.08	0.97 ± 0.82	− 0.91 ± 0.71		
Female	247 (47.6%)	257 (53.6%)	237 (47.2%)	262 (52.6%)	242 (49.9%)	259 (50.3%)	125 (48.3%)	144 (53.5%)
Male	271 (52.2%)	222 (46.3%)	264 (52.6%)	236 (47.4%)	239 (49.9%)	256 (49.7%)	133 (51.4%)	125 (46.5%)
Non-binary	1 (0.2%)	0	1 (0.2%)	0	1 (0.2%)	0	1 (0.4%)	0
18–25 years	22 (4.2%)	58 (12.1%)	22 (4.4%)	56 (11.2%)	20 (3.5%)	57 (11.1%)	8 (3.1%)	40 (14.9%)
26–40 years	114 (22.0%)	96 (20.0%)	84 (16.7%)	121 (24.3%)	85 (18.1%)	125 (24.3%)	45 (17.4%)	66 (24.5%)
41–64 years	254 (48.9%)	179 (41.1%)	241(48.0%)	196 (39.4%)	224 (47.9%)	212 (41.2%)	127 (49.0%)	94 (34.9%)
> 65 years	129 (24.9%)	146 (30.5%)	155 (30.9%)	125 (25.1%)	153 (30.5%)	121 (23.5%)	79 (30.5%)	69 (25.7%)
Underweight	15 (2.9%)	13 (2.7%)	6 (1.2%)	22 (4.4%)	10 (1.8%)	18 (3.5%)	4 (1.5%)	12 (4.5%)
Normal weight	153 (29.5%)	204 (42.6%)	88 (17.5%)	269 (54.0%)	124 (24.2%)	232 (45.1%)	44 (17.0%)	148 (55%)
Overweight	162 (31.2%)	159 (33.2%)	173 (34.5%)	153 (30.7%)	145 (24.4%)	182 (35.3%)	73 (28.2%)	82 (30.5%)
Obese	182 (35.1%)	98 (20.5%)	230 (45.8%)	48 (9.6%)	199 (49.6%)	75 (14.6%)	136 (62.9%)	23 (8.6%)

*Abbreviations: DC* Danger Control, *FC* Fear Control, *CA* Caries, *DB* Diabetes, *OW* Overweight. “n” represents the number of observations. “%” refers to the percentage of the total sample

The sample size for the different control processes of the three diseases differs slightly from the overall sample size: *n* = 998 for Caries (for BMI variables *n* = 986). *n* = 997 for Diabetes (for BMI variables *n* = 985). *n* = 1,000 for Overweight (for BMI variables *n* = 989). *n* = 259 for danger control process of all three diseases at the same time (for BMI variables *n* = 257). *n* = 269 for fear control process of all three diseases at the same time (for BMI variables *n* = 265)

### Multinomial Logit (MNL)

The approach to creating the final model was successive, and four MNL models are shown in Table 7. These build on each other and were supplemented step by step with additional explanatory variables. As a basic model (model 1), the influence of the warnings (= health consequences: caries, diabetes, overweight), the symbols “Shock” and “Stop” as well as “Price”, and the no-buy option were examined. When it comes to interpreting the effects of the warnings (Overweight, Diabetes, Caries), the results are set in reference to the base alternative (no symbol, no warning, no other attribute). When it comes to the symbols (Shock, Stop, No symbol (= text-only)) and the symbol-warning interactions, the “text-only” attribute served as a reference-category, to describe the additional effect of Shock/Stop vs. text-only. This was done because we were especially interested in finding out how the graphic additions compare to a warning text without an image. Warnings claims solely, have been studied in different contexts already e.g. [14, 28, 58]. However, the efficacy of a combined text–picture approach, like the one tested in Tobacco, is seldom combined in food choice and has not yet been related to different concepts of fear/danger control. In the second model, additionally, the interactions between the warning symbols and the diseases were examined (Shock-Overweight, Shock-Diabetes, Stop-Diabetes, Stop-Caries). In the third model, socio-demographic aspects were added to the interactions between the warning symbols and diseases. These were additional interactions of the warning effects with age, as well as interactions of the previously formed factors of the PCA (factors 1 and 3) with the warnings to elucidate their specificity for the selective target audience. Finally, in the fourth model, interactions between the danger control processes of the diseases and the warnings were added. The final estimated model (model 4) exhibited a good model fit (McFadden R^2^ = 0.276), hence depicting an explanatory value of roughly 58% in R^2^, which is high.

**Table 7:** MNL model analysis

Attributes	Model 1		Model 2		Model 3		Model 4	
	**Estimate**	**z-value**	**Estimate**	**z-value**	**Estimate**	**z-value**	**Estimate**	**z-value**
**Warnings**								
Overweight	0.39*	5.21	0.10	1.76	0.01	0.09	0.03	0.44
Diabetes	− 0.18*	− 3.04	− 0.34*	− 2.40	− 0.43*	− 2.99	− 0.36*	− 2.41
Caries	− 0.07*	− 2.04	− 0.57*	− 4.73	− 0.67*	− 5.45	− 0.63*	− 4.89
**Symbol-Warning-Interactions**								
**Shock**^**a**^**/**Shock-OW	− 0.79*^a^	− 15.74	− 0.86*	− 11.93	− 0.89*	− 12.13	− 0.96*	− 12.19
Shock-DB			− 0.92*	− 7.10	− 0.95*	− 7.18	− 1.03*	− 7.53
**Stop**^**a**^/Stop-DB	0.27*^a^	4.27	0.07	0.56	0.06	0.46	0.00	0.02
Stop-CA			0.49*	4.62	0.49*	4.57	0.45*	4.10
**Fixed effects/ASC**								
Price	− 1.97*	− 32.91	− 1.92*	− 25.88	− 1.91*	− 25.89	− 1.91*	− 24.22
No-buy	− 1.45*	− 12.93	− 1.69*	− 19.34	− 1.69*	− 19.34	− 1.77*	− 18.27
**Socio-demographics/PCA**								
Age-Shock-OW					− 0.01*	− 2.26	− 0.01*	− 2.10
Age-Shock-DB					0.00	0.62	0.01	1.74
Age-Stop-DB					− 0.01*	− 5.40	− 0.01*	− 5.60
Age-Stop-CA					− 0.01*	− 7.42	− 0.01*	− 6.83
Factor 1 – OW					0.12*	4.36	0.10*	3.32
Factor 3 – OW					− 0.12*	− 4.30	− 0.11*	− 3.92
**EPPM**								
Stop – DC DB							0.10*	3.64
Stop – DC CA							− 0.02	− 0.83
Shock – DC DB							0.16***	3.50
Shock – DC OW							− 0.21***	− 4.50
**Log-Likelihood**	** − 12,523**		** − 12,518**		** − 12,155**		** − 10,918**	
**McFadden R**^**2**^	**0.170**		**0.170**		**0.1955**		**0.2766**	

*Abbreviations OW* Overweight, *DB* Diabetes, *CA* Caries, *DC* Danger Control

Significance: *p < 0.05. **a** = For the first model no symbol-disease interactions were calculated, but only the symbols “Stop” and “Shock” were used. Reference category: For “Warnings” we refer to the base alternative as reference. For “Symbol”/”Symbol-Warning-Interaction” we used the “text-only” attribute as reference-category, to describe the additional effect of Shock/Stop vs. text-only

Model 1 shows that when it comes to the warnings, there are differences between the three health consequences. We see that the warnings concerning caries and diabetes significantly reduced the probability of buying chocolate or gummy bears (caries: ß =  − 0.07*; diabetes: ß =  − 0.18*), whereas the warning concerning overweight showed a positive effect (overweight: ß = 0.39*). This tendency can be observed across all four models. Thus, when it comes to the warnings themselves (without graphical addition), diabetes seems to have the most deterrent effect. In the case of the symbols per se (without text addition of the disease), we found that the shock image had a negative influence (Shock: ß =  − 0.79*), while the stop sign had a positive influence (Stop: ß = 0.27*).

Since we were interested in seeing how the warnings interacted with additional graphical symbols, we added interactions between the graphical symbols and the health consequences to the model (model 2). The results show that the interactions with the shocking pictures (in combination with overweight and diabetes) significantly reduced the probability of buying the products (Shock-Overweight: ß =  − 0.86*, Shock-Diabetes: ß =  − 0.92*). Even though the overweight warning showed a weak positive effect, in combination with the respective shocking picture we found a negative effect on the buying decision. Regarding the interactions with the stop signs, we observed opposite results. The interaction between the stop sign and diabetes showed no significant effect (Stop-Diabetes: ß = 0.07). The interaction between the stop sign and caries showed a significant positive effect (Stop-Caries: ß = 0.49*). This supports the assumption that some graphical additions can strengthen the effect of text-only warnings, but the effect seems to vary depending on the type of graphical addition.

When it comes to the socio-demographic variables (age) that were additionally included in model 3, we found that with increasing age of the participants, the Shock-Overweight warning had a more important negative effect on the choice of the products (Age-Shock-Overweight: ß =  − 0.01*). With increasing age, also the stop sign reduced the purchase probability, and a negative effect was measured when the sign was combined with caries and diabetes (Age-Stop-Caries: ß =  − 0.01*; Age-Stop-Diabetes: ß =  − 0.01*).

The perceptions of participants concerning HWM (factors 1 and 3) in general were considered as well. Although the two factors “ineffectiveness of warning labels” (factor 1) and “uselessness of warning labels” (factor 3) combined rather negative attitudes toward warning labels, they nevertheless point in different directions when combined with the warning about overweight. The interaction between factor 1 “ineffectiveness of warning labels” and overweight had a positive effect on the product choice (factor 1-Overweight: ß = 0.12*). However, the interaction between factor 3 “uselessness of warning labels” and overweight had a negative effect on the latter (factor 3-Overweight: ß =  − 0.12*). This indicates that a high agreement with statements that HWM are useless because one knows the ingredients of the products and makes purchase decisions based on them, still negatively influences a purchase of the offered products. On the other hand, the positive influence of statements that HWM are ineffective because they will quickly lose their effect, and a negative attitude towards public health intervention may be an indication for mistrust or reactance.

Finally, interactions between the danger control processes of the diseases and the graphical symbols “Stop” and “Shock” were part of model 4. The results show that with increasing severity of the danger control process for overweight, the shocking pictures reinforced the negative effects on choice (Shock-DC Overweight: ß =  − 0.21*). Hence, reduced the likelihood for choice. However, with increasing severity of the danger control process for diabetes, the shocking pictures had no reinforcing negative effect but a positive effect on the product choice (Shock-DC Diabetes: ß = 0.16*). The same was true for the stop signs and an increasing severity of the danger control for diabetes (Stop-DC Diabetes: ß = 0.10*). However, the interaction between the stop sign and increasing severity of danger control for caries points in a negative direction, although the effect was not significant (Stop-DC Caries: ß =  − 0.02). This result suggests that the effectiveness of the symbols also depends on the diseases the warning is about. Which may have to relate to the perceived severity, closeness, and proximity to the disease labeled and perceived self-efficacy in regard to changing the mal-consumption behavior. The results of all four models also show that a higher price and the option to choose no product (no-buy option) significantly decrease the likelihood of consumers buying one of the products (price model 4: ß =  − 1.91*, no-buy model 4: ß =  − 1.77*). More so, results show that drastic shock pictures (in combination with a text message, that eases to engage in a danger control process), may be more effective with regard to preventive health behavior. In all four models, the shock-warning-interaction was negative.

## Discussion

The main aim of this study was to examine how different types of health warning messages in combination with graphical applications affect consumer choices of sweets, in order to better understand their impact on consumer behavior. The results show that, when it comes to the health consequences addressed by the warnings per se (without graphical addition), it appears that the warnings about caries and diabetes have a deterring effect but need to be understood in their relation to fear and danger control processes and may need a sensitive approach to target populations. The warning about becoming overweight suggests an effective influence only if combined with graphical warnings. One explanation for this could be that being overweight might be least perceived as a “disease”. Overweight is highly prevalent in the German population. In 2019, the average overweight rate in Germany was 54% [47]. It might be that people consider it “normal” to be overweight and do not consider the chronic conditions to be serious problems. Another reason might be that being overweight is a multidimensional process that is additionally promoted by other factors in addition to excessive sugar consumption, e.g., lack of physical activity. At the same time, the disease presents itself less suddenly and has more of a long-lasting effect, which is therefore diffused and not as easy to trace for people, whereas immediate health crises have an effect on dietary behavior change [48]. From tobacco warnings, we can assume that depicting short-term external health effects as opposed to longer-term chronic diseases may be more effective because they occur more immediately [49, 50]. This could be the case for caries, which occurs comparatively quickly and, moreover, is directly visible or perceptible. Additionally, it seems to be relatively easy for a person to prevent the disease by maintaining regular oral hygiene.

Regarding the interactions between the different diseases and the additional graphical images, we find two phenomena: The interaction between the diseases and the shocking images confirms what has been previously shown in the literature: Warnings that combine images and text have a greater impact than text-only warnings [15, 51, 52]. In our case, a shocking image enhances the effect of a text-only effect in relation to various diseases (overweight and diabetes).

A new discovery, however, is that in our case the graphic addition of a red stop sign did not have the same effect. The stop sign in combination with the diabetes warning showed a non-significant positive effect, the combination of “stop” and “caries” even showed a significant positive effect; indicating a higher likelihood to oversee the intended negative consumption adherence. This is contrary to what we would have expected from the literature, even though we know that symbolic warnings are less effective than graphical images [49, 53]. There were several aspects that favored the use of a red stop sign. First of all, warning labels must catch the consumer's attention within seconds and convey the relevant information. Symbols and colors that are already associated with certain concepts in society through repeated exposure are suitable and recommended for this [54, 55]. In this regard, the red octagon is associated with a stop and danger [54, 55]. Then, red warning labels have been reported to be noticeable and associated with unhealthful products [56, 57], even though a black color seems to require less time to be detected by consumers [55, 57]. Additionally, a stop sign-like symbol (black and white octagon) is already being successfully and effectively used as a text-based health warning in South America [58]. Based on this, we would also have expected a negative impact on consumption behavior, but maybe it even induces reluctance as of an information overload. Another explanation for the positive effects could nevertheless still be that the warning with the supplementary stop sign was misunderstood by some participants. It is possible that the message was understood exactly the wrong way round, i.e., in the sense of: “This chocolate stops caries”. This association could arise, for example, if the adjacent text warning was not read or not read completely. Particularly in combination with the caries text warning, the chocolate or gummy bears, for example, were perhaps considered to be without sugar or reduced in sugar and could therefore reduce or stop caries. This could eventually explain the strong positive effect of caries for this interaction; however, we cannot find evidence for similar situations in the literature.

Regarding interactions with socio-demographic characteristics, it is interesting to see that with increasing age, especially the warning about being overweight but also the addition of a stop sign does have a significant negative influence on the likelihood of choosing sweets. At first glance, this seems contrary as the risk of becoming overweight is a warning in itself, and the interactions with “stop” in the model as such did not show a significant or a positive effect. Against the background of age, however, the explanation of this effect could lie in life experience. With regard to overweight, people are more likely to be affected with increasing age, and the long-term effect of excessive consumption may then become more present [59]. One study that related to the knowledge of health consequences of SSBs, showed that with increasing age, more people know that obesity can be a consequence of excessive sugar consumption [60]. Regarding the stop symbol, it is possible that older people have more experience with the symbol in the sense that they have known the symbol for longer and have internalized its meaning.

Regarding the interactions between the diseases and the factors from the PCA that dealt with the participants’ perception of the HWM, agreement with the statements summarized under factor 1 (“ineffectiveness of warning labels”) in combination with the warning about overweight has a positive influence on the likelihood of choosing the sweets. This factor combines statements that are rather negative toward warnings or demonstrate that the consumer does not believe in their success. In contrast, agreement with the statements summarized under factor 3 (“uselessness of warning labels”) in combination with the warning about obesity leads to a negative effect, although this factor also summarizes statements that consider warnings to be superfluous. The difference between the factors and their different effects could lie in the fact that factor 3 rejects warnings due to sufficient knowledge (“I don’t need warning labels because I shop very consciously”). In contrast, in factor 1 a general aversion to government intervention (“The government is trying to exert more and more influence, but not with me!”) is more likely to be the reason for rejection. Increased knowledge about healthy food behavior and the consequences of excessive sugar consumption can contribute to lower consumption rates, e.g., of SSBs [61].

The EPPM was introduced to explain the effect of fear appeals and to evaluate the message effects. The distribution across the three health threats with regard to the two processes was very similar. For “caries”, “diabetes”, and “overweight”, the segmentation into danger control and fear control was almost 50%. We can conclude from this that none of the health threats was able to trigger a more perceived threat than the others. Concerning the interactions between the warning symbols and the danger control processes for the different diseases, it can be seen that the effects depend on the disease that the warning addresses. Although the three diseases we presented are among the top three categories of diseases that consumers associate with sugar consumption [33], presumably consumers differently assess the risk and the severity of the diseases regarding the different effects of short-term vs. long-term consequences of overconsumption. An individual who is in the danger control process for a disease knows effective strategies to avert the “danger”. Using warning messages could be one strategy. It explains that when in the danger control process for overweight (and caries), the shocking picture might reinforce the decision not to choose this product, as the association between sugar consumption and overweight is very clear and is also clearly depicted on the associated shocking image. One could expect a similar effect for diabetes, but this is not the case. It seems as if there is more insecurity regarding the disease diabetes, possibly because the disease is less visible than overweight at first sight and thus feels further away from one’s own control. Also, there is less knowledge about diabetes and its symptoms or consequences [62]. This could be due to the fact that there is also very little research on the public knowledge and risk perception for developing diabetes [62, 63]. These might be reasons why neither a corresponding shock image nor a stop sign had a reinforcing negative effect when it comes to the danger control process of diabetes.

In our models, also the price of the products played an important role. The fact that a higher price has a negative influence on the purchase probability is not surprising, but it underlines once again the effect that, for example, the introduction of a sugar tax could have on the purchase of sweets or sweet drinks [64]. In addition to the warning, the high price creates a quasi-double inhibition.

### Limitations

There are limitations to this study. The participants of this study were recruited via an online panel provider. The quotas set insure a representation of the German population with regard to gender, age, level of education, and net household income. However, quota sampling doesn’t allow for a random selection, which means that our sample could be affected by selection and information biases, e.g., because people without internet access couldn’t take part in this study. As with any model, the application of the discrete choice method is a simplification of the complex reality. The setting of the choice experiment was artificial, involving hypothetical selection decisions using images of products and warning labels. Furthermore, the respondents made their decisions on the basis of the attributes and characteristics presented to them. In a real purchase decision, the attributes of the products are usually not available to the buyer in the same systematic form, and within the choice experiment, the respondents were exposed to the health warnings longer than in a real shopping situation. However, this allowed us a clear differentiation between the different aspects (design, message) of health warning messages to better understand their efficacy, evaluation, and reactance with regard to their impact on consumers’ behavior.

Due to the hypothetical nature of the choice situation, no real price had to be paid for the selected products. This circumstance may also have led to result distortions.

Additionally, it needs to be mentioned that some of the respondents might already have diabetes. This information was not collected in the questionnaire. According to current research, there is a positive correlation between BMI and type 2 diabetes [65]. The present sample contains a high proportion of overweight people (> 60%). Compared to the German population, our sample shows a higher number of individuals with a BMI over 25 [47]. For these reasons, diabetes might have had a more deterrent effect compared to caries and overweight.

When it comes to the statements for the EPPM, we need to mention that the scales for querying the model-related items were proportionally lengthy. Every respondent had to rate a total of 36 items, with 12 items referring to one health threat. Each item had to be evaluated three times, and each time only the health threat changed. In retrospect, a division of the sample into three groups, with every group evaluating one health threat, could have led to more informative results. When it comes to the results of the PCA, it needs to be mentioned that factor 1 “ineffectiveness of warning labels” had a comparably low scale reliability (factor 1 = 0.487), however content-wise, the items of this resulting factor formed a consistent component with an explanatory variance of 12,45%.

## Conclusion

The results of the study have shown that emotional graphical images combined with text health warning labels might be one interesting approach to counteract the increasing spread of sugar consumption-related effects on health. In this context, shocking pictures are particularly noteworthy, as they showed the most promising outcomes regarding the non-purchase of sweets in this study setting. The findings also suggest that combining graphical elements with text warnings requires a thorough approach because the health effects of immediate (caries) and more distant health consequences (diabetes/obesity) influence how warning messages are perceived. This is equally true for the different types of graphical design additions (shocking pictures vs. stop signs). Policy-makers should therefore be aware of the meaning of implicit or already learned (cultural) symbols. It is necessary to weigh up and test the effect of new types of claims, especially against the background that consumers are used to positive labels, which means that implementing negative labels or claims might require different approaches. Presumably, the connection to the non-purchase is already better learned or understood for shocking pictures due to the implementation on cigarette packings. Future studies should take this into account. Against this background, it is also worth mentioning that although this study did examine health warning messages that trigger negative emotions, next studies are encouraged to examine the effect of positively formulated health warnings (e.g. humor). Furthermore, future studies should study health warning messages in the context of other public health policies, such as a sugar tax. It is known from policies in the tobacco context, that increasing taxes on tobacco products is one of the most effective strategies, even compared to the health warning messages. As it is surprising to see how the perception of different diseases and their consequences impacts the severity of the warning, we see a need to catch up on the perception and the meaning or understanding of NCDs in the population in the context of a targeted health policy. In addition, as older people are a vulnerable group for obesity and diabetes, there seems to be the need of specific prevention targets for particular age vulnerability. Further studies within the public health sector need to put forward new scientific evidence to help political decision-makers in their future decisions.

## Data Availability

The datasets used and analyzed during the current study are available from the corresponding author on reasonable request.
